# Supplementary material for: HDAC1 SUMOylation promotes Argonaute-directed transcriptional silencing in C. elegans
Source: eLife. 2021 May 18;10:e63299. doi: 10.7554/eLife.63299 (PMC8131101; doi:10.7554/eLife.63299)
Supplement: Supplementary file 1. [file elife-63299-supp1.docx]

**Supplementary File 1.** Summary of RNAi-based genetic screen of chromatin factors and modifiers using a silenced piRNA sensor.

| RNAi | Score | Number | Description |
| --- | --- | --- | --- |
| L4440 | 0% | N=20 | Empty vector |
| *nrde-2* | 64% | N=28 | piRNA pathway |
| *nrde-4* | 14% | N=28 | piRNA pathway |
| *set-25* | 13% | N=23 | piRNA pathway |
| *hda-1* | 14% | N=28 | NuRD complex |
| *chd-3* | 6% | N=34 | NuRD complex |
| *lin-53* | 12% | N=25 | NuRD complex |
| *let-418* | 19% | N=26 | NuRD complex |
| *sin-3* | 30% | N=27 | SIN-3 complex |
| *dcp-66* | 8% | N=25 | HDA-1 interactor |
| *lin-40* | 6% | N=34 | HDA-1 interactor |
| *spr-5* | 8% | N=24 | CoREST |
| *isw-1* | 11% | N=27 | SWI/SNIF complex |
| *ssl-1* | 9% | N=23 | SWI/SNIF complex |
| *set-33* | 17% | N=24 | histone methyltransferase |
| *cbp-3* | 13% | N=24 | histone acetyltransferase |
| *hda-2* | 9% | N=22 | histone deacetyltransferase |
| *lin-61* | 5% | N=38 | Chromatin binding |
| *mrg-1* | 12% | N=52 | Chromatin binding |
| *lin-49* | 8% | N=24 | Bromodomain protein |
| *trr-1* | 8% | N=25 | Transcription coregulator |
| *din-1* | 10% | N=20 | transcriptional repressor |
| *gmeb-3* | 11% | N=18 | transcription coactivator |
| *taf-1* | 21% | N=19 | TATA-box binding |
| *taf-5* | 10% | N=21 | TATA-box binding |
| *air-2* | 56% | N=25 | Kinase |
| *psr-1* | 7% | N=29 | arginine demethylase |
| *acin-1* | 10% | N=30 | RNA binding |
| *smo-1* | 28% | N=54 | SUMO |
| *ubc-9* | 29% | N=58 | SUMO E2 |
