# Supplementary material for: HDAC1 SUMOylation promotes Argonaute-directed transcriptional silencing in C. elegans
Source: eLife. 2021 May 18;10:e63299. doi: 10.7554/eLife.63299 (PMC8131101; doi:10.7554/eLife.63299)
Supplement: Supplementary file 4. [file elife-63299-supp4.docx]

**Supplementary File 4.** List of gRNA and ssOligo donor sequences

| guide | sequence | gene | usage |
| --- | --- | --- | --- |
| oYD274 | CATAATTTTGTCGAGCAAGT | wago-9 | **gfp::tev::2xflag::degron**::wago-9 |
| oYD217  oYD218 | GCTATTTCGGAGATCCGCTC  CATTCCGAAGCGAAACTTCT | wago-9  wago-9 | wago-9 **deletion mutant** |
| oYD426 | ccatcaaacATGAACTCAAA | hda-1 | **3xflag::degron**::hda-1 |
| oYD422 | TGAAGAAGATCCGTCTTTAG | let-418 | **3xflag::degron**::let-418 |
| HK_smo-1_sgRNA #5 | gagactcccgctataaacgA | smo-1 | **3xflag**::smo-1 |
| HK_ubc-9_crRNA #11 | AAGGATACGATTTGGGAAGG | ubc-9 | ubc-9[**G56R**] |
| HK_hda-1_crRNA1 | GCTCAGTTTGAGTCGGAAGG | hda-1 | hda-1[**K444R**] |
|  |  |  | hda-1[**K459R**] |
|  |  |  | hda-1[**K444R, K459R**] |
|  |  |  | hda-1::**gfp** |
| HK_hda-1_cpf1_crRNA1 | CTCTGTCTTCTGACGCTTTTC | hda-1 | hda-1::**his10::smo-1[GG to AA]** |
| HK_hda-1_cpf1_crRNA2 | GTGTTTTACTCTGTCTTCTGA | hda-1 |  |
| HK_hda-1_cpf1_crRNA3 | CTCCGTACGCTGACGCTTTT | hda-1(KKRR) | hda-1[K444R,K459R]:**his10:::smo-1[GG to AA]** |
| HK_hda-1_cpf1_crRNA4 | GTGTTTTTACTCCGTACGCT | hda-1(KKRR) | hda-1[K444R,K459R]::**gfp** |
| HK_mrg-1_crRNA 1 | TTCCTTTGAAGACATctga | mrg-1 | **2xflag::degron**::mrg-1 |
| HK_mep-1_sgRNA #4 | GCGCAAAAGAAGGAAGACGG | mep-1 | mep-1::**gfp::degron** |
| ssOligo sequence | | | usage |
| TACTCGTATTATCGAAATCGTCACTTCACAAAAGCTCTAAtcccgagattctctcttttattgtcacgtattc | | | wago-9 **deletion** |
| CCAGGGAGAAAGGATACGATTTGGGAAGGCCGGTTATACAGAGTATGTGAAGCTAGAATTCGG | | | ubc-9[**G56R**] |
| GAGCGTGAGGGAGATGATCGAAGAAACGAGAGCGATGCGCGCAGAGCAGCTCAGTTTGAATCAGAGGGTGGTGAAAAGCG  TCAGCGTACGGAGTAAaacactaaaatgtgccgccgtcgaattcag | | | hda-1[**K444R**]  hda-1[**K459R**]  hda-1[**K444R, K459R**] |
